# Impact of parental migration on psychosocial well-being of children left behind: a qualitative study in rural China

**DOI:** 10.1186/s12939-018-0795-z

**Published:** 2018-06-15

**Authors:** Chenyue Zhao, Feng Wang, Xudong Zhou, Minmin Jiang, Therese Hesketh

**Affiliations:** 1 Institute for Global Health, University College London, 30 Guilford St, London, WC1N 1EH UK; 2 Department of Child and Adolescent Psychiatry, New York University School of Medicine, 1 Park Ave, New York, NY 10016 USA; 3 Institute of Social Medicine, Zhejiang University, 866 Yuhangtang Rd, Zhejiang, 310000 Hangzhou China; 4 Department of Public Policy and Law, Zhejiang Normal University, 668 Yingbin St, Jinhua, Zhejiang, 321004 China; 5 Centre for Global Health, Zhejiang University School of Medicine, 866 Yuhangtang Rd, Zhejiang, 310000 Hangzhou China

**Keywords:** Migration, Left-behind children, Psychosocial well-being, Parent-child relationship, Family structure, Family functioning, Attachment

## Abstract

**Background:**

Tens of millions of rural “left-behind children (LBC)” in China grow up experiencing prolonged separation from their migrant worker parents. This study aimed to explore how children are affected by parental migration, from the perspectives of children, parents, and grandparents, focusing on the experiences of prolonged parent-child separation and relationship dynamics in the extended family.

**Methods:**

Qualitative in-depth interviews were conducted in a migrant-sending rural area of eastern China. Participants included 25 children (aged 7 to 14), 17 parents, and 13 grandparents, from 30 families, as well as 24 key informants from the communities. Data analysis followed a grounded theory approach.

**Results:**

The results showed that despite the original purpose of benefiting children, parental migration resulted in challenges in child psychosocial well-being, due to the emotional impacts from prolonged parent-child separation. Parental absence also led to inadequate care and support for left-behind children. The negative effects of parental migration may be exacerbated by other vulnerabilities such as parents’ divorce, poverty and grandparent caregivers’ frailty. Concerns about child well-being made some migrants decide to return home permanently, because of the altered trade-offs of migration.

**Conclusion:**

Prolonged separation following migration often disrupts parent-child relationships and results in psychosocial difficulties in LBC, especially among those who live with multiple adversities in the family. Community-based interventions may help migrant parents and co-resident caregivers to better engage children and promote their resilience.

## Background

In many parts of the world, labor migration has had profound impacts on family structures and the home communities of migrants [1]. Due to stringent entry policies, financial constraints, and limited access to public goods at the destination, migrants are often forced to leave their children behind in their home country or hometown for lengthy periods of time [2]. The number of these so-called “left-behind children” (LBC) is high in many low- and middle-income countries. In China, massive rural-urban migration has driven the number of left-behind children (LBC) up to 61 million, accounting for 38% of children in rural China [3].

Despite the economic benefits generated by labor migration, parental absence may lead directly to decreased care, stimulation and supervision [4]. The non-traditional family structures may be risks for future psychopathology in children [5], and exacerbate the vulnerabilities of children with less social support [6, 7]. Substantial evidence has demonstrated the psychological impact from parental absence. A meta-analysis found that parental migration was associated with increased risk of mental health problems in Chinese children [8]. LBC were also shown to be prone to loneliness [9–11], low life satisfaction and depression [12, 13], low self-esteem [14], and behavioral problems [15, 16], in China and globally.

As lengthy physical presence and high quality of care with strong emotional investment are key factors in maintaining attachment relationships [17], during long periods of parent-child separation, children of all ages may develop negative emotions and feelings that disrupt attachment bonds [18]. A study in the Philippines demonstrated children’s feelings of discomfort, inability to communicate, and ambivalence about relationships with the migrant father [4]. Pribilsky [19] suggested the depression-like disorder (*nervios*) in Ecuadorian left-behind children might be explained by disruptions to parent-child attachment.

In the absence of one or both parents, the quality of care arrangements in the left-behind family is crucial to the impact of parental migration on children. Effective psychosocial support from co-resident caregivers may help children cope with parental absence [20]. Some quantitative studies suggest that LBC living with one parent had better psychological well-being than those living only with grandparents [21, 22], and children in the care of grandparents fared better than those cared for by other relatives, in China and Southeast Asia [21, 23, 24]. Dreby’s qualitative study in Mexico [12] found that many LBC received inadequate care from co-resident caregivers, which led to behavioral and academic difficulties. Yet existing qualitative studies rarely examined the caregiving environment provided by both migrant parents and co-resident caregivers, and how different caregivers may affect and respond to children’s psychosocial risks.

Although migration in this study is within one country, the rural-to-urban migrants in China are similar to transnational migrants in terms of socioeconomic incentives and barriers to securing family welfare at the migration destination [2], due to the household registration system in China and the fundamental urban-rural divide in the country [25]. Our study aimed to explore how children are affected by parental migration, from the perspectives of children, parents, and grandparents, with a focus on the experiences of prolonged parent-child separation and relationship dynamics in the extended family. The emerging concepts from our qualitative data would be tested against a coding paradigm proposed by Strauss & Corbin [26] to generate theory and hypotheses.

## Methods

### Study area and participants

This was a qualitative study comprising in-depth interviews with left behind children, their migrant parents and co-resident caregivers in a rural migrant-sending area in Kaihua County, Zhejiang Province, China. Located in an inland mountainous area far from the developed coastal cities, Kaihua is one of the poorest counties in the province, with a population of 350,000. The study was conducted with the support of the local county branch of the Women’s Federation, a government agency responsible for children’s welfare issues, with representation from central government to village level.

Depsite the so-called One Child Policy, in some areas of China such as Kaihua, a second child is allowed if the first is a girl. In the absence of one or both parents, grandparent almost always help care for children as a virtual expectation in Chinese culture [27]. According to an unpublished survey by Kaihua Women’s Federation, about 40% of school-age children in the county are left behind by one or both parents. In the first stage of sampling, 12 villages, all from different townships (out of 18 townships in Kaihua) were selected, to achieve geographical representativeness and population coverage across the county. Based on information obtained from the Women’s Federation staff and village committee members, the second stage involved a purposeful sampling approach to include children and their left-behind families of diverse socioeconomic status, care arrangements and other characteristics. In the third stage, additional participants were recruited when new categories were discovered, and when relationships between categories needed to be verified to formulate the overall storyline. We interviewed children with family members present if this was preferred by the caregiver. The families would offer validation and emotional support when necessary, although their presence might also have prevented the child from disclosing some true feelings. However, many interviews with children were conducted separate from their caregiver, because of limited availability of family members during the field visit.

Qualitative in-depth interviews were conducted with 25 children, 17 parents, and 13 grandparents, from 30 families. Children ranged in age from 7 to 14 (mean = 10.9, SD = 1.8). The family composition and migration patterns are shown in Table 1, with each row representing a family. Two key informants in each village were also interviewed to explore community contexts and any additional perspectives in relation to the aim of this study. These included a total of 15 village leaders, and 9 other community members.

**Table 1:** Demographics, family structure, and migration pattern of interviewed migrant families

Index child			Co-resident adult	Migrant parent	Child age when left behind
Sex	Age	Sibling			
Girl	10	Elder sister	Mother	Father only	2
Boy	12	Younger sister	Mother	Father only	10
Girl	11	None	Other relatives	Both parents	5
Girl	11	None	Grandparents	Both parents	2
Girl	9	Elder sister	Mother & Grandma	Father only	Shortly after birth
Girl	10	Younger brother	Mother & Grandparents	Father only	Unknown
Boy	10	None	Grandma	Both parents	3
Girl	14	Elder sister	Mother	Father only	Shortly after birth
Boy	12	None	Grandparents	Both parents	4
Boy	13	Younger brother	Grandparents	Both parents	1
Girl	8	Younger brother	Grandparents	Both parents	Shortly after birth
Boy	14	None	Grandparents	Both parents	1
Boy	11	None	Grandparents	Both parents	A few months
Girl	11	None	Grandparents	Both parents	3
Boy	11	None	Grandpa	Both parents	4
Girl	12	Younger brother	Mother	Father only	6
Girl	10	Elder sister	Mother	Father only	5
Girl	9	None	Grandparents	Mother only	9
Girl	7	Elder sister	Grandparents	Both parents	3
Boy	12	None	Grandma	Both parents	5
Girl	10	None	Grandparents	Both parents	4
Boy	12	None	Grandparents	Both parents	5 or 6
Boy	13	None	Grandma	Father only	11
Boy	13	None	Grandparents	Both parents	3
Boy	10	None	Mother & Grandma	Father only	1
Boy	12	None	Mother	Father only	Unknown
Girl	12	Elder sister	Mother & Grandparents	Father only	Shortly after birth
Boy	9	None	Grandma	Both parents	4
Girl	13	None	Grandparents	Both parents	7
Boy	8	Elder brother	Grandparents	Both parents	5

### Interview procedure

Interviews were conducted during September to October 2013, and from January to February 2014, around Chinese New Year when most migrant workers return home for the celebrations. All interviews were conducted by the first author in Mandarin. Each interview lasted for about 20 to 40 min. The selected families were visited at home to recruit children, parents and/or caregivers who were present. Children who were not at home were recruited at the local community center where they usually spend time after school. Interviews with migrant parents from these families were conducted in the second study period.

Ethical approval was received from both the Ethics Review Committee of University College London and Zhejiang University prior to the field study. The study purpose, the interview process and topics were explained to all participants, and informed written consent was obtained from all interviewees, including a parent or caregiver of all children. Participants were guaranteed that non-participation in the study would not affect any benefits from the community or government, and the interview would stop at any time if any child participant or caregiver felt uncomfortable to continue. We had also made arrangements to provide a local psychological counseling service, through accessing an initial hotline, for any interviewees who felt they wanted to further explore areas of difficulty. No one except the researchers had access to the personal information and interviews documented in this study. Pseudonyms were given to the participants who are quoted or mentioned in this article.

Interviews were audio-recorded and transcribed verbatim with specific consent from the participants. Observational notes were also taken on interviewees’ behaviors and facial expressions, as well as interactions when multiple family members were interviewed conjointly. Interview questions covered topics including incentives and concerns about migration, experiences of and feelings toward separation, communication with the migrant parent(s), and care and support from the caregiver at home. The questions were adapted to the child’s age, and rephrased when examining the adult family members’ perspectives.

### Analysis

A respectful yet critical stance was adopted when the theoretical and empirical literature was reviewed. For the present study, the analysis of interview data followed principles of the grounded theory approach [26] and was jointly conducted by the first and last authors. Both authors constantly reflected on their preconceptions developed while engaging with literature and coding the data, before incorporating any concepts into the narrative of this study. Data were gathered and analyzed simultaneously, and continually checked and revised throughout the research process, including modifications to interview questions, as new theoretical ideas emerged.

**Fig. 1:**
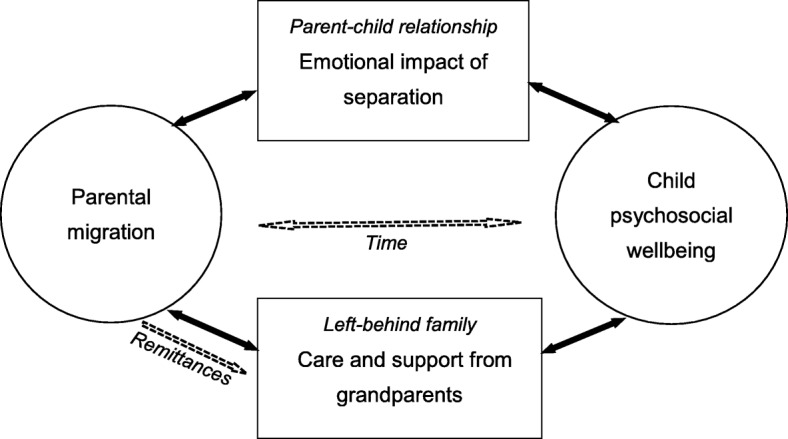
Conceptual framework of key impact mechanisms of parental migration on child psychosocial well-being

Perspectives of multiple family members were triangulated where possible, to improve understanding of the dynamics in the extended family. We followed the principles of open, axial, and selective coding [26]. First, all transcript data were open coded to define the preliminary categories and their dimensions. Memos were taken to generate explanations of the emerging concepts, and to further develop the key categories, as well as the relationships between them. This process helped the authors to become sensitive to the meanings without forcing explanations on the data. Then in the axial coding phase, logical and meaningful connections among the most salient categories were made, with reference to a predetermined paradigm model that involved the context, conditions, relationships and coping mechanisms in response to the separation, and the consequences of these strategies [26]. Lastly, in selective coding, data analysis and memo-writing became increasingly conceptual, through constant comparison between incidents and categories from the data. A core variable, experiences of migration and disrupted family dynamics, eventually emerged. The ground theory paradigm and the results of this study informed a conceptual framework (Fig. 1), also drawing on the socio-ecological model of human development [28] and a family stress model [20].

## Results

### Perceptions on the rationales of migration

Our interview results suggest that the primary incentive for migration was perceived as “to earn more money”, by both adults and children in the families. Migration represented the best chance of survival and possible prosperity of the family, especially for the children. Parents felt “there’s no other way” but to migrate to the cities. A mother illuminated this rationale vividly:

*We have to leave because there are no jobs here except farming. Their dad and I could have stayed, but we really need to earn money just for the kids, (to pay for) their high school or college. We’re not very young now, and we won’t be making much money when we get old.*

Some older children had positive perceptions of the reasons for their parents’ migration. A 12-year-old boy said, “I do miss him (father). I wish he could come home more often and play with me and spend more time together, but I do understand that he has to go and earn money.” In another case, the migrant mother reported how her 10-year-old son coped:

*He knows that the family couldn’t survive without the money, so we have to go... He understands this and has become mature. Sometimes he says, “Mom, next year can you move back home?” But after a few words to soothe him, he is fine with it.*

Interestingly there was no clear distinction in economic status between migrant and non-migrant families in Kaihua, according to the local community leaders. This relates to the various dynamics of migration, that is, in many cases adults from the poorer families migrate and improve their economic status, while better-off adults may choose not to migrate, leading to greater economic equality among residents.

### Emotional consequences of separation from migrant parents

We examined experiences and perceptions of children that may indicate their level of psychosocial well-being, especially in relation to their separation from migrant parents. Children’s emotional distress in relation to parental migration, for example, loneliness, sadness, and frustration, were reported by children themselves, parents, and caregivers. Different patterns of attachment and interactions with parents also reflected children’s perceptions of migration and disrupted family relationships. In most cases the parents were aware of the effects of migration on their children, and attempted to closely monitor the emotional and behavioral well-being of the children, at home or over distance.

#### Attachment feelings and emotional distress

Most migrant parents were only able to reunite with their children during Chinese New Year and school holidays in the summer. The majority of children said they miss their parents a lot. Despite the lengthy absences of parents, some children maintained strong emotional attachments with the parents, through many occasions of separation and reunion. The feelings seemed most intense when migrant parents were about to leave home again after a brief reunion. For example, a 13-year-old boy Zhuang reported that he “felt pain” or “would cry” at his parents’ departure, and “wished they could spend more time at home” with him. His mother described the situation, “When he sees us, he’s really happy, and when we have to leave again, he becomes sad and upset.”

An interview with another family showed how the effects of migration were perceived. Both the son, Xing (aged 12), and the daughter, Jing (aged 10), were clearly emotionally affected by their father’s migration. The father, Qiang, had been away for two years, leaving the children with their mother, Huan. The couple explained the challenges for the parent-child relationships:

*Huan: Kids always want their dad to come home; Xing always says on the phone, “Dad, when are you coming home? I miss you so much.” Jing is a bit quiet though. I say to them the best thing you can do is to study harder. Your dad is (working for) your future.*

*Qiang: When I miss the kids I call and chat; when it’s not busy at work I visit home - I come back four or five times a year. Although we talk on the phone, they’re not that close to you when you don’t come home often – they only say the good stuff, and I’m also careful not to say things which will hurt. When we’re home together we’re more frank with each other and I get to know how they feel.*

We found that when parent-child separation occurred after early childhood, the adverse effects may be limited. Some children like Xing, for example, tended to openly express their feelings towards parents’ migration and were even able to persuade them to spend less time away. Yet many children, especially girls, seemed to internalize their distress when coping with the separation. Rui, an 11-year-old girl, lived with her aunt, and both her parents worked in the provincial capital. She was interviewed soon after a short reunion in the summer holidays when she had been to see her parents in the city:

*I miss my parents very much. Especially in these days when the school just started, it feels like everything I see reminds me of the time I spent with them. Then I sometimes cry a little. I read a book or something to help me stop thinking about them.*

With clear emotional attachment, Rui expressed both affection and sadness even when she was talking about her difficult feelings towards the separation from her parents. The fact that Rui was not separated from her parents before she went to elementary school may have strengthened the attachment bonds and her abilities in coping with the separation. However, it was notable that four children actually cried during the interview when asked about their feelings towards their migrant parents, illustrating how painful the separation was. Nine-year-old Hong was interviewed with her mother, Lian. The father was working in a province thousands of kilometers away, and the family was very poor. Hong started weeping as soon as her father was mentioned and her interview had to be suspended.

#### Ambivalent attitudes towards parents and frustration about the separation

In contrast, some children seemed ambivalent about their migrant parents, even showing indifference during interactions with the parents. Most of these children were left behind in early childhood, some during infancy. The emotional connections and attachment relationship were likely to be considerably weakened by the extended separation. Shengli, the father of an 11-year-old boy named Yi, left home shortly after his son’s birth. Four years later, his wife joined him in Hangzhou city, leaving the child behind with grandparents. Shengli explained:

*When we are away my son doesn’t really communicate with me, although I call him quite often. Sometimes grandma has to make him pick up. Over the phone he sounds annoyed sometimes, especially when we [he and his wife] urge him to study hard. But when I’m home - usually 3 or 4 times a year - he gets more clingy and tells me about things that worry him.*

Shengli’s accounts showed his son’s ambivalent feelings towards him. Yi himself said in an interview later, “I wish I could see him (Dad) more but I also feel a little uncomfortable because he spends little time at home with us.” In comparison, some children seemed more detached from migrant parents, and even reunions were not desirable. Ten-year-old Zhimin’s parents migrated to a coastal city shortly after his birth, leaving him with his two grandparents. After a few years, the parents owned a grocery store in the city, and organized for Zhimin to go to kindergarten there, but the boy refused. The child was interviewed together with his grandmother:

*Interviewer: Do you like chatting with your parents over the phone?*

*Zhimin: No. They just make a fuss, and only talk about study.*

*Interviewer: So do you miss your mom and dad?*

*Zhimin: Sometime I do, sometimes I don’t.*

*Grandma: It’s just that I was the one who brought him up since he was born. When his parents are back home once or twice a year, he doesn’t even seem close to them. It especially frustrates him at the point when his parents go back to the city again.*

This was one example of frustration in children about the separation from parents, especially after a brief reunion. We found that it might be more difficult for vulnerable families with complex problems to manage the parent-child relationship stress after migration. An example was Xia, aged 14, whose mother, Fang, re-married after her first husband died, and had Xia in her late 30s (very late in rural China). Xia’s father had been a migrant worker since his teenage years, so she was cared for by Fang from birth. Xia talked about her experience and feelings about her father:

*Xia: I don’t talk to people much, or hang out with others…I stay in my house after school.*

*Interviewer: Do you often talk with your dad?*

*Xia: I have nothing to say to him. I usually hang up within one minute when he calls. I used to get along very well with him, when I was little - like two persons in one. But as I grew up we grew apart. I don’t really miss him now.*

*Fang: I asked her dad to call up every week and have a chat with her. But she has nothing to say to him. Even when he’s back at home it doesn’t get much better.*

Towards the end of the interview Xia became tearful, but she settled quickly, and was able to continue the conversation. Adverse effects from her father’s migration and complex disruptions in family relationships appeared to cause considerable emotional challenges for Xia. We later learned from Fang that Xia had been receiving psychiatric treatments for a year. Poverty in the unstable family may have added to the overall difficulties facing Xia. With no extra help from grandparents, Fang had to rely heavily on the government’s social assistance in raising Xia.

### Challenges in grandparents’ child care

Care and support in the left-behind family is essential to protect children from risks for psychosocial well-being due to parental absence. In fact, many children became much closer to caregiver grandparents than to parents. A Women’s Federation staff member quoted the Chinese proverb “closeness between every other generation” to describe this situation. However, some grandparents were quite frail, and almost none were able to offer academic guidance (all grandparents in this study were illiterate or semi-literate). Interviewees also described the “generation gap”, as the huge discrepancies between the grandparents and children, resulting from massive social change in China over the past decades. Overall, the grandparents did not fully replace the parents’ responsibilities or roles in a traditional family structure or attachment relationship. As a 10-year-old boy named Tengfa described:

*I feel alright (about my parents’ absence) because my grandparents take care of me well. But I like my parents better, because they understand more about what I usually think about and what I usually do, and they give me better advice and support when I need those.*

The quality of grandparents’ care was found to be especially important when children suffered from multiple adversities in the family and demonstrated psychosocial difficulties. Besides parental migration, additional family adversities such as parents’ divorce and grandparent caregiver’s frailty, make children more prone to well-being problems. This was shown by a 13-year-old boy Dongyang. His parents got divorced when he was eight. His father was a construction worker who would often travel to nearby rural areas, while his mother had migrated to Quzhou, a city near Kaihua. Dongyang started to cry during an interview that had to be suspended. Later we talked to his neighbor, a grandmother who knew Dongyang’s family well and occasionally cared for him. The neighbor reported:

*It’s his 80-year-old (paternal) grandma who mainly cares for him (grandpa passed away), after his dad won the custody. But grandma is a bit too frail as a caregiver, because sometimes she actually needs help from the child. And the money sent from his dad is barely enough. Now even the child’s bed is broken and hasn’t been fixed...You can see he’s quite short for his age. He rarely appears to be happy…Occasionally I invite him for dinner at our house, as he sometimes plays with my grandson.*

### Child well-being difficulties in the left-behind family

Children’s well-being issues under the inadequate care environment at home usually led to parents’ concerns. Some parents even decided to return home permanently to provide better childcare, when they found the child to be persistently unhappy, lonely, or showing signs of a dysfunctional relationship with caregivers. Yilin, mother of an 11-year-old boy, recently quit her job in the city and moved back to work in a local factory. She left home when her son was six, following her husband who migrated shortly after the child was born. Yilin described the situation:

*When I’m home it’s better…at least he chats with his dad on the phone. When I’m away he is certainly lonely. He would often say, ‘I’m so bored being alone, Mom.’ Sometime when he goes out after finishing homework, the other kids are already gone. Now that I’m home, I sometimes invite kids to come and play with him. That didn’t happen when I wasn’t around.*

The boy’s grandmother said she could not handle her grandson, who would not listen to her or communicate with her much, and would often make an excuse to go out. Consequently, the child’s loneliness did not relieve until his mother’s return. Some grandparents in our study had difficulties in communication with the children and in disciplining them, which raised the parents’ concern. The mother of a vivacious 11-year-old girl named Tinging described:

*Tingting is really too lively and can’t stay focused…Her grandparents are unable to communicate with her and discipline her much. Nowadays the older generation can’t really understand children’s thoughts and behaviors, or their problems. I worry that Tingting will start puberty soon, and her grandparents’ old values and standards won’t be helpful anyways.*

She later said that she and her husband had decided to look for jobs near home, because they were worried about Tingting’s overall development in their prolonged absence. In some other cases, child behavioral problems became concerning when stricter measures adopted by grandparents appeared to cause conflicts within the left-behind family. A migrant mother named Xiaojin was worried about her son’s recent change in attitudes and behaviors against grandparents’ disciplining. While her husband would continue working in the city, Xiaojin had decided to quit the city job and come back home next year to take care of the 11-year-old. As she said:

*It’s just his temper that I’m really worried about. He’s become very rebellious in the last few months. He doesn’t listen to his grandparents at all, and always answers back when grandparents point out what he did wrong or tell him what to do. When I’m home it’s better. With us being away, his grandparents can’t control him, although they try very hard.*

The above-mentioned situations showed that children’s emotional and behavioral difficulties made migrant parents modify original migration plans. Although we found no clear evidence regarding the long-term impact of return migration, the decision to return was always intended to benefit overall child well-being, while taking into account the possible economic challenges.

## Discussion

Very few qualitative studies published in English or Chinese have addressed the psychosocial well-being issues of left-behind children in China. Results from this study offer crucial insights into the phenomenon of parental migration and how children are affected in the disrupted family structure. The substantive conceptual categories that emerged from our data were: perceived economic benefits of migration for children, emotional impacts from disrupted attachment with parents, challenges in grandparents’ child care, and child well-being difficulties in the left-behind family. Relating to all these categories, the core variable, experiences of migration and disrupted family dynamics, reflected the central aspects of our conceptual framework and accounted for most of the variation in the characteristics of migrant families. The key concepts and the connections between them formulated the hypotheses in our grounded theory: 1) despite the original purpose of benefiting children, parental migration leads to child well-being difficulties, due to the emotional impacts from prolonged separation, and the inadequate care and support in the absence of parent(s); 2) the negative effects of parental migration may be exacerbated by other adversities such as poverty, parents’ divorce, or grandparent caregiver’s frailty, and 3) concerns about child well-being made some migrants decide to return home permanently, because of the altered trade-offs of migration, as the child grows or certain worrying events occur.

While the impact of parental migration on child well-being involves complex mechanisms, a direct consequence is the parent-child separation that leads to emotional impacts on children, as indicated by the pathway at the top of our conceptual framework. The restructured relationship dynamics revolve around the children’s response to the separation, and the migrant parents’ challenge to provide care remotely. Existing qualitative studies often focused on children’s thoughts and feelings as psychological outcomes [12, 13], rather than the perceptions of both parents and children in relation to their relationship functioning.

Attachment theory indicates that long-term caregiver absence may result in emotional distress for children regardless of age [17]. Our results indicated that thoughts of their migrant parents made many children feel sadness, even demonstrating depressive symptoms. Those who were separated from parents in later childhood appear to have stronger attachment bonds and emotional resilience. In comparison, some children who were left behind during infancy, such as Zhimin and Xia, tend to hold back their emotions when interacting and communicating with migrant parents. With ambivalent attitudes towards parental absence in a dysfunctional parent-child relationship, these children demonstrate frustration about the separation from parents, while the parents indicate discomfort about their ambiguous roles as remote caregivers. These cases support theories of attachment [29] as well as existing evidence which shows that early separation may be particularly damaging to psychological well-being [30]. Hoang and Yeoh [31] found in rural Vietnam that some LBC intentionally distanced themselves from their parents. Other qualitative studies [12, 13, 32] suggested that ineffective communication styles between migrant parents and children undermined child well-being.

Given the loss of physical proximity and the insecure attachment with parents, effective care arrangements and adequate support in the left-behind family are crucial (illustrated as another pathway shown in Fig. 1), especially when children experience well-being challenges. Additionally, children who are exposed to vulnerabilities other than parental migration, such as parents’ divorce and extreme poverty, are in need of extra care and support from the extended family, or even the community. Negative events were found to cause higher levels of stress in LBC than in non-LBC [33]. Grandparents’ care may be particularly critical in protecting the disadvantaged LBC from crisis situations, as demonstrated in a study in Ghana [34]. In our study, although grandparents are willing to make great efforts in childcare and become primary caregivers who manage children’s daily life, they are often unable to fulfill the challenging roles in protecting children from risks to psychosocial well-being. The generation gap, which is especially relevant for China because of the huge sociocultural discrepancies between the generations, may be a barrier to effective communication and emotional connections with children, and thus may hinder grandparents’ dedication. Also, the physical strengths of grandparents are often limited; some of them have even become so frail that the older children appeared to be taking care of them more than the reverse.

As child well-being concerns increase, and grandparents become older, some migrant mothers decide to move back home, often to address the current or potential risks to child development. The negative impact of lengthy separation from parents on children tends to increase over time, as exemplified by Xia’s case. A longitudinal study in China has shown a decline of happiness and social support during LBC’s early adolescence, but not among non-LBC [35]. The decision of return migration is made despite significant loss of household income, which suggests that parents fear their absence is defeating the original purpose of migration and may negatively impact their children’s well-being. The parent-child reunion is likely to be protective for child psychosocial well-being when the family structure and functioning are restored.

### Limitations

The small sample in this study was recruited from a relatively wealthy province in China, and hence cannot be nationally representative, considering the massive underdeveloped rural areas across the country. Although we aimed at recruiting families with diverse contexts in terms of family structure, socioeconomic status, and migration timing, the small sample did not allow us to systematically investigate all variables in family contexts and distinguish their individual effects on child well-being. We were also unable to compare the specific roles of migrant fathers and migrant mothers in attachment relationships, or to comprehensively examine gender disparities in child well-being. In addition, despite our efforts to build trust during the conversations, some participants, especially young children, may have been unable to fully express their feelings in the context of research. Finally, as is the case with interviewing volunteers in any setting, some of the most vulnerable children with the more extreme experiences may have avoided being interviewed.

## Conclusion and study implications

Our findings showed that prolonged separation following migration often disrupted parent-child relationships and resulted in emotional difficulties in children. The emotional impacts may lead to psychosocial risks particularly among left-behind children living with multiple adversities in the family. In vulnerable left-behind families, grandparents’ care may be inadequate to meet the needs in child development and well-being. Both migrant parents and co-resident caregivers need additional support in better engaging children and promoting their resilience.

In our study sites, our research has contributed to the development of two intervention programs: an intervention to help parents communicate with children over distance and deal with reunion and the point of separation, and a community-based program to provide care and support to LBC in “Children’s Clubs” [36]. These interventions should continue to be developed in rural China and potentially adapted in other low-resource contexts globally, to benefit the massive number of families living apart due to migration. Future research may establish systematic intervention mechanisms and evidence on improvements in child well-being outcomes.

## Abbreviations

LBC: Left-behind children
